# Preparation of Janus-Structured Evaporators for Enhanced Solar-Driven Interfacial Evaporation and Seawater Desalination

**DOI:** 10.3390/gels11050368

**Published:** 2025-05-17

**Authors:** Junjie Liao, Luyang Hu, Haoran Wang, Zhe Yang, Xiaonan Wu, Yumin Zhang

**Affiliations:** 1 School of Materials Science and Engineering, Anhui University of Science and Technology, Huainan 232001, China; 17355451848@163.com (J.L.); hrwang2013@163.com (H.W.); 2 Anhui Industrial Generic Technology Research Center for New Materials from Coal-Based Solid Wastes, Huainan 232001, China; yangzhe010120@163.com (Z.Y.); 13839318581@163.com (X.W.); 3 National Key Laboratory of Science and Technology on Advanced Composites in Special Environment, Harbin 150001, China; zhym@hit.edu.cn

**Keywords:** Janus-structured evaporator, interface evaporation, solar desalination, hydrogel, salt resistance

## Abstract

Solar-driven interfacial evaporation has emerged as a sustainable and highly efficient technology for seawater desalination, attracting considerable attention for its potential to address global water scarcity. However, challenges such as low evaporation rates and salt accumulation significantly hinder the performance and operational lifespan of evaporators. Here, we present an innovative Janus-structured evaporator featuring distinct operational mechanisms through the integration of a hydrophobic PVDF-HFP@PPy photothermal membrane and a hydrophilic PVA-CF@TA-Fe^3+^ hydrogel, coupled with a unidirectional flow configuration. Distinct from conventional Janus evaporators that depend on interfacial water transport through asymmetric layers, our design achieves two pivotal innovations: (1) the integration of a lateral fluid flow path with the Janus architecture to enable sustained brine replenishment and salt rejection and (2) the creation of dual vapor escape pathways (hydrophobic and hydrophilic layers) synergized with hydrogel-mediated water activation to elevate evaporation kinetics. Under 1 sun illumination, the evaporator achieves a maximum evaporation rate of 2.26 kg m^−2^ h^−1^ with a photothermal efficiency of 84.6%, in both unidirectional flow and suspension modes. Notably, the evaporation performance remains stable across a range of saline conditions, demonstrating remarkable resistance to salt accumulation. Even during continuous evaporation of highly saline water (10% brine), the evaporator maintains an evaporation rate of 2.10 kg m^−2^ h^−1^ without observable salt precipitation. The dual anti-salt strategies—enabled by the Janus structure and unidirectional flow design—underscore the evaporator’s capability for sustained high performance and long-term stability in saline environments. These findings provide valuable insights into the development of next-generation solar evaporators that deliver high performance, long-term stability, and robustness in saline and hypersaline environments.

## 1. Introduction

In the contemporary era, rapid population growth and extensive industrialization are driving a significant yearly increase in global water consumption. Given that the majority of the Earth’s water resources are contained within oceans, there is an urgent need for technologies capable of seawater desalination to ensure a sustainable water supply [1,2,3,4,5,6]. Conventional desalination methods, including distillation and reverse osmosis, demand significant fossil energy inputs and rely on complex infrastructure, posing substantial economic and environmental challenges [7,8,9,10]. As a clean, renewable energy source, solar energy offers considerable potential for desalination through solar-driven interfacial vapor generation, which leverages photothermal conversion principles, providing a sustainable means for water treatment and desalination [11,12,13,14,15,16]. The efficiency of solar-driven interfacial evaporation systems relies on the optimization of light absorption, heat management, and water transport. Selecting suitable photothermal materials and designing an efficient evaporator structure are crucial to achieving high steam generation rates. Commonly employed photothermal materials are categorized into carbon-based materials, inorganic semiconductors, organic small molecules, and polymer materials, each of which has been extensively applied across different system designs [17,18,19,20,21,22,23].

However, the interfacial evaporation of seawater often leads to salt precipitation, which diminishes the light absorption area, obstructs pores, disrupts water transport, and significantly impairs evaporation performance. A commonly adopted strategy to mitigate salt crystallization involves segregating the functional zones of the evaporator, thereby spatially separating evaporation and salt precipitation regions [24,25,26,27]. For instance, Xia et al. designed an evaporator comprising a horizontal evaporation disk and a vertical suction cotton thread, which creates a radial concentration gradient from the disk center to its edges. This innovative configuration ensures that salt crystals form only at the periphery of the disk, preserving the central evaporation zone [25]. Another approach utilizes non-contact evaporation, as demonstrated by Zhang’s team. Their design integrates water with a non-contact solar evaporator, creating a wickless and confined water layer where natural convection facilitates salt discharge, effectively preventing salt accumulation at the evaporation interface [26]. Additionally, Janus structures have been explored, in which evaporators with multilayer configurations and asymmetric wettability properties are employed. In such designs, salt is confined to the hydrophilic layer, preventing its deposition on the hydrophobic photothermal interface and substantially improving the stability of the evaporation process [27].

Salt transport can also be facilitated through controlled brine flow driven by convection and diffusion arising from concentration gradients. Yang’s team demonstrated this concept with a three-dimensional desalination evaporator featuring vertically aligned mass transfer bridges, enabling brine diffusion via concentration differences [28]. Similarly, Yuan and colleagues developed an evaporator incorporating a dual-mode water exchange structure, combining in-plane diffusion and in-tube migration to achieve continuous and uniform water transfer [29]. The Marangoni effect, driven by surface tension gradients resulting from salinity concentration differences, offers yet another mechanism to address salt precipitation. This effect directs the brine flow from regions of lower surface tension to those of higher surface tension, aiding in salt discharge and maintaining a stable evaporation interface [30,31,32]. By leveraging unidirectional fluid evaporation driven by these flow-based salt transport mechanisms, evaporators can achieve long-term operation without salt clogging. Moreover, unidirectional flow anti-salt deposition evaporation is broadly compatible with most photothermal materials, circumventing the need for complex structural modifications and expanding the applicability of this technique [33,34].

The evaporation performance of solar-driven evaporators can be further enhanced by modifying the state of water within the evaporator, encompassing bound water, intermediate water in the transition zone, and free water [21]. Among the most commonly utilized materials for this purpose are hydrogels. Poly(vinyl alcohol) (PVA) hydrogel, characterized by its three-dimensional network structure, has emerged as a promising material for solar energy evaporators, particularly when combined with various photothermal materials. Hydrogels are typically synthesized through physical (non-covalent) or chemical (covalent) cross-linking, resulting in a robust and versatile structure. The hydrogel matrix facilitates the storage of water within its interconnected network, effectively altering the state of water to lower its enthalpy of evaporation. This reduction enhances the efficiency of the evaporation process. Moreover, the hydrogel’s excellent hydrophilicity and strong capillary action ensure continuous and efficient water transport to the evaporation surface, enabling sustained and rapid evaporation rates. These properties, combined with its compatibility with advanced photothermal materials, underscore the hydrogel’s potential as an indispensable component in next-generation solar evaporator designs [35,36,37].

In this study, we report the successful design and fabrication of a novel Janus-structured evaporator that integrates a hydrophobic PVDF-HFP@PPy membrane and a hydrophilic PVA-CF@TA-Fe^3+^ hydrogel–cellulose fabric, addressing key limitations of conventional hydrogel-based or Janus systems. The unique material configuration provides distinct advantages: (1) In situ formation of tannic acid–iron (TA-Fe^3+^) complexes within the hydrogel matrix circumvents nanoparticle agglomeration issues common in composite hydrogels, ensuring stable photothermal performance. (2) TA-Fe^3+^ and polypyrrole (PPy) have been shown to exhibit excellent photo- and heat-conversion properties, making them highly effective for solar-driven applications [9,10,17,18]. The integration of these materials within a double-layer architecture further amplifies light absorption by optimizing the interaction between the photothermal material and incident solar energy. (3) The Janus design coupled with unidirectional water transport achieves simultaneous salt rejection and sustained evaporation, overcoming the trade-off between salt accumulation and water replenishment observed in traditional hydrogel evaporators. Crucially, the hydrogel component reduces evaporation enthalpy by modulating bound water states—a feature absent in non-hydrogel Janus systems—while the hydrophobic layer prevents salt deposition without compromising vapor escape. The dual anti-salt strategies—Janus structuring and unidirectional flow—introduced in this work provide valuable insights for the development of high-performance solar evaporators capable of sustained operation in saline environments.

## 2. Results and Discussion

### 2.1. Characterization of the Janus-Structured Evaporator

**Figure 1:**
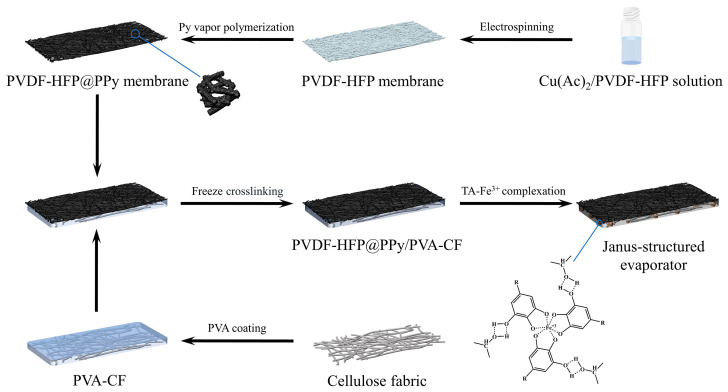
Schematic illustration of preparation of Janus-structured evaporator.

**Figure 2:**
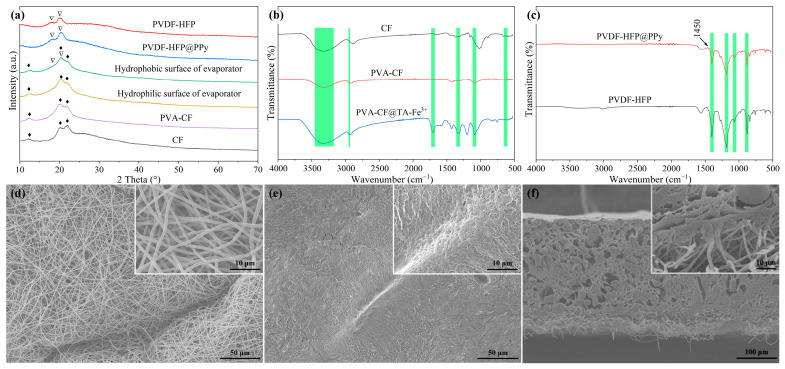
Composition and microstructure of samples. (**a**) XRD patterns. FTIR spectra of (**b**) hydrophilic surface and (**c**) hydrophobic surface. SEM images of (**d**) hydrophobic surface, (**e**) hydrophilic surface, and (**f**) cross-section of Janus-structured evaporator.

The fabrication process of the Janus-structured evaporator is illustrated in Figure 1. First, a PVDF-HFP@PPy membrane was prepared through electrospinning, followed by an in situ reaction with pyrrole (Py) vapor. Simultaneously, a PVA solution was infiltrated into cellulose fabric to obtain PVA-CF. Subsequently, the PVDF-HFP@PPy membrane was assembled onto the PVA-CF surface. Next, three cycles of freeze–thaw crosslinking wre performed, after which an in situ deposition of a tannic acid-Fe^3+^ complex was conducted to achieve the Janus membrane with dual-functional interfaces. To examine the evolution of the phase structure, the XRD spectra of the samples were analyzed (Figure 2a). For the PVDF-HFP nanofibrous membranes, distinct diffraction peaks are observed at 18.3° and 20.0°, corresponding to the coexistence of the semi-crystalline α-phase and polar β-phase of PVDF-HFP. This dual-phase structure likely results from the electrostatic spinning process, which promotes phase formation under specific processing conditions, as corroborated by previous studies [38,39]. Upon the deposition of PPy onto the PVDF-HFP membrane to form PVDF-HFP@PPy, no characteristic peaks of PPy are detected in the XRD spectra. The absence of PPy diffraction peaks is likely due to its low concentration, which falls below the detection threshold of XRD. In contrast, the CF exhibits three distinct diffraction peaks at 12°, 20°, and 22°, corresponding to its intrinsic crystalline structure. Following the PVA gelation treatment, the XRD patterns of the PVA-CF composite show no additional peaks, consistent with the amorphous nature of PVA. After TA-Fe^3+^ complexation, the diffraction patterns of the hydrophilic surface of the Janus-structured evaporator remain unchanged, closely resembling those of the PVA-CF composite. On the hydrophobic side of the evaporator, the XRD patterns not only retain the characteristic peaks of PVDF-HFP but also prominently display the diffraction peaks associated with CF. This confirms the successful integration of the PVDF-HFP layer while maintaining the underlying structural features of CF.

To further elucidate the structural evolution of the Janus-structured evaporator surface, FTIR spectroscopy was employed. As illustrated in Figure 2b, prominent peaks for CF are detected at approximately 1020 cm^−1^, 2900 cm^−1^, and 3330 cm^−1^. Upon the combination of CF with PVA, a characteristic peak at 1087 cm^−1^, corresponding to the stretching vibration of the C-O bond in PVA, emerges in the PVA-CF composite. Following treatment with TA and Fe^3+^, the FT-IR spectrum of PVA-CF@TA-Fe^3+^ reveals significant peak broadening centered at ~3300 cm^−1^. This broadening originates from the formation of hydrogen bonds between TA and PVA [40], which not only promotes intermolecular crosslinking but also enhances the hydrophilicity of the material. Furthermore, a peak at 613 cm^−1^, corresponding to the stretching vibration of the Fe-O bond, and a peak at 1705 cm^−1^, associated with the carbonyl group in PVA-CF@TA-Fe^3+^, are detected [10]. Additionally, the bending vibration of the C-O bond in TA produces a peak at 1339 cm^−1^, while peaks at 2944 cm^−1^ and 2910 cm^−1^ correspond to the C-H stretching vibration and the asymmetric stretching vibration of -CH_2_, respectively [35]. These peaks are present in both the PVA-CF@TA-Fe^3+^ and PVA-CF samples, further confirming the successful complexation of TA and Fe^3+^ with PVA [10,41]. The FT-IR spectra of the hydrophobic surface are presented in Figure 2c. The characteristic peaks of PVDF-HFP are observed at 1402 cm^−1^, 1186 cm^−1^, 1072 cm^−1^, and 878 cm^−1^, corresponding to -CF stretching, -CF_2_ asymmetric stretching, C-C stretching, and -CF_3_ stretching vibrations, respectively [42,43]. These peaks are retained in PVDF-HFP@Ppy, signifying that the structural integrity of PVDF-HFP is maintained post deposition. Additionally, the appearance of a characteristic peak around 1450 cm^−1^, attributed to the C-N stretching vibration of Ppy, indicates the successful incorporation of Ppy onto the PVDF-HFP nanofibers [44]. Importantly, no new functional groups are observed in PVDF-HFP@Ppy, confirming that the deposition of Ppy do not alter the fundamental chemical structure of PVDF-HFP.

Figure 2d–f illustrate the representative micromorphology of the Janus-structured evaporator. The hydrophobic surface exhibits a distinctive reticular structure, similar to that of the PVDF-HFP membrane (Figure 2d and Figure S1), but with noticeable wrinkling caused by freeze–thaw expansion–contraction cycles; this phenomenon enhances the efficient binding of hydrophilic and hydrophobic layers while improving light absorption through intensified multiple surface reflections. Polypyrrole (Ppy) nanoparticles with a diameter of less than 1 μm are uniformly distributed on the surface of the PVDF-HFP nanofibers, facilitating both hydrophobicity and light absorption. On the hydrophilic surface, a dense array of micropores is visible (Figure 2e), which establish hierarchical vapor transport channels within the evaporation layer, enabling unimpeded steam diffusion. Meanwhile, the surface is adorned with a substantial deposition of TA-Fe^3+^ nanoparticles. A cross-sectional view of the evaporator is depicted in Figure 2f and Figure S2. The fibrous network of the cellulose fabric is distinctly observable (Figure S3), with fibers uniformly embedded within the hydrogel matrix. The structure presents abundant macropores, which, together with the robust integration between the PVDF-HFP@Ppy nanofibers and the hydrophilic layer, ensure efficient water and energy transport.

### 2.2. Mechanical Property and Wettability of the Janus-Structured Evaporator

**Figure 3:**
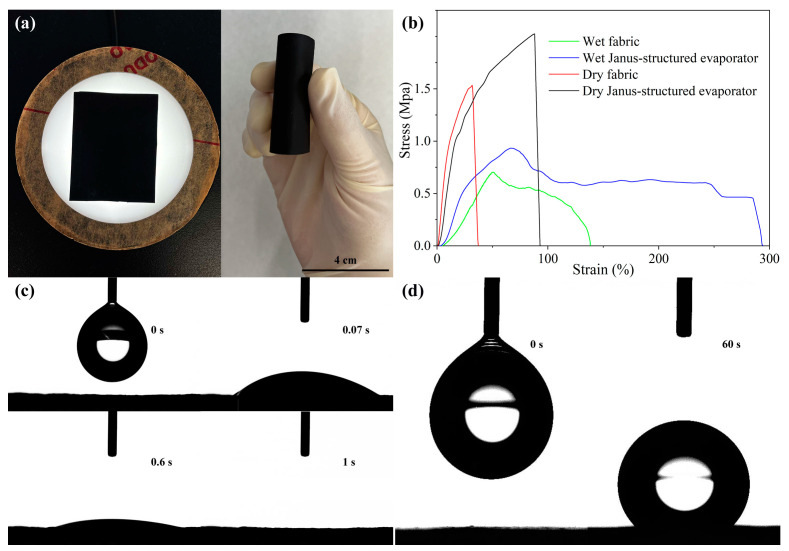
Mechanical properties and wettability of the samples. (**a**) Photograph and bending image of the Janus-structured evaporator. (**b**) Stress–strain curves of the evaporator compared to the original fabric. Evolution of water contact angles over time for the (**c**) hydrophilic surface and (**d**) hydrophobic surface of the evaporator.

Figure 3a presents a physical photograph and bending test image of the Janus-structured evaporator, highlighting its excellent flexibility under manual bending. The evaporator’s pliability underscores its potential for practical applications, where mechanical adaptability is often required. To further evaluate its mechanical properties, a stress–strain test was conducted, as shown in Figure 3b. The results reveal that in the dry state, the tensile strength of the evaporator significantly surpasses that of the original cellulose fabric, indicating an enhancement in mechanical robustness following structural modifications (Figure 2f and Figure S3). In the wet state, the evaporator retains superior tensile strength and tensile deformation compared to the original fabric, demonstrating its resilience and adaptability under hydrated conditions. These findings demonstrate the improved mechanical stability of the Janus-structured evaporator, which is essential for prolonged operational durability in water-based environments.

The wettability of the evaporator plays a critical role in influencing its evaporation performance. To assess this, the dynamic diffusion behavior of water droplets on the hydrophilic surface of the Janus-structured evaporator was observed, as illustrated in Figure 3c. Upon contacting the hydrophilic surface, a 4 μL water droplet exhibited instantaneous spreading, with the dynamic contact angle rapidly decreasing to approximately 0° within 1 s. In comparison, the PVA-CF membrane required 2 s to reach a similar contact angle under identical test conditions (Figure S4). This behavior highlights the superhydrophilicity of the PVA-CF@TA-Fe^3+^ layer, which can be attributed to the synergistic effects of hydrophilic functional groups, photothermal particles, and the enhanced capillary action resulting from the reduced pore size in the evaporator. In contrast, the hydrophobic characteristic of the opposing surface is depicted in Figure 3d. As expected, the water droplet maintains a substantial contact angle of approximately 130° even after 1 min, whereas the contact angle of the original PVDF-HFP membrane is shown to be 116.5° (Figure S5), demonstrating the pronounced hydrophobicity of the PVDF-HFP@PPy nanofiber membrane. This hydrophobic characteristic is essential for maintaining efficient water transport on the hydrophilic side while preventing salt accumulation on the photothermal layer, thereby enhancing overall evaporation performance.

### 2.3. Evaporation Performance for Pure Water

**Figure 4:**
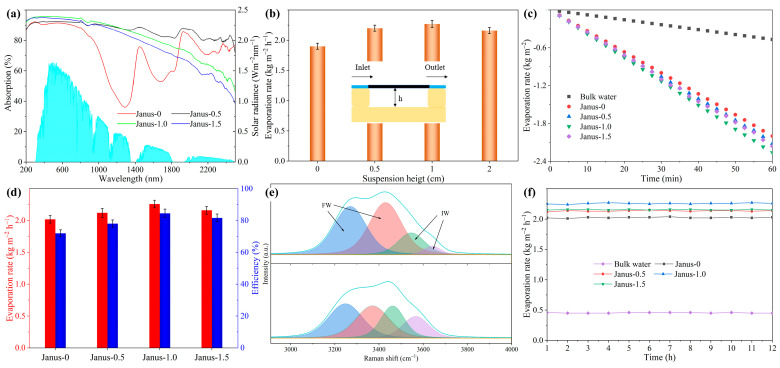
The interfacial evaporation performance of the Janus-structured evaporator for pure water. (**a**) UV-vis-NIR absorption spectra of the evaporator, along with the normalized spectral solar irradiance density of the air mass 1.5 global (AM 1.5 G) tilt solar spectrum. (**b**) Water evaporation rates for evaporators suspended at heights ranging from 0 to 2 cm under simulated sunlight at an intensity of 1.0 kW m^−2^. The inset illustrates the schematic representation of the unidirectional flow system and suspension mode used for water evaporation. (**c**) Mass loss curves for bulk water and water within the evaporator under simulated AM 1.5 G solar spectrum illumination. (**d**) Evaporation rate and energy conversion efficiency of the evaporators. (**e**) Raman spectra of the water and Janus-structured evaporator. (**f**) Evaporation stability tests for various evaporators over a continuous 12 h.

Efficient solar light absorption is a critical factor of evaporation performance in photothermal systems. To quantitatively evaluate the absorption characteristics, the optical absorbance spectra of the samples were measured using a UV-VIS-NIR spectrometer equipped with an integrating sphere. As depicted in Figure 4a, the Janus-structured photothermal evaporator demonstrates an absorbance exceeding 90% across the solar spectrum (AM 1.5 G, see Note S1). This high absorbance can be attributed to two primary factors: (1) the effective photon trapping by photothermal nanoparticles embedded within the membrane’s surface (Figure 2d) and (2) the unique double-layer structure of the evaporator (Figure 2f), which promotes multiple internal reflections of light. Among the samples, Janus-1.0 and Janus-1.5 exhibit slightly higher absorbance, owing to the increased thickness of their hydrophobic layers (Figure S6), which further augments light reflection and retention within the surface [27,45,46]. In addition to absorption, energy losses during the evaporation process—including heat conduction, convection, and thermal radiation—directly impact the efficiency of solar-to-vapor conversion. Optimizing structural design and thermal management can minimize these losses. To explore these effects, a unidirectional flow suspension method was employed for water evaporation experiments, as shown in Figure 4b. The influence of the suspension height (0–2 cm) on the evaporation rate was investigated. The evaporation rate increased from 1.90 kg m^−2^ h^−1^ at 0 cm to a peak of 2.26 kg m^−2^ h^−1^ at 1 cm, before declining to 2.16 kg m^−2^ h^−1^ at 2 cm. These results indicate that a suspension height of 1 cm provides optimal thermal management, reducing heat loss through thermal radiation and conduction. Unless otherwise stated, subsequent experiments were conducted at this optimal height.

Thermal performance was further analyzed by monitoring temperature changes under solar illumination. Figure S7 illustrates the rapid temperature increase of the Janus-1.0 photothermal evaporator compared to bulk water over 5 min of illumination. The Janus-1.0’s surface temperature increased from 18 °C to ~ 33 °C within the 50 s and stabilized thereafter (Figure S8), while bulk water exhibited only a modest temperature rise of 1–2 °C. The rapid heating of the evaporator is attributed to its high light-to-heat conversion efficiency and low heat loss. To assess vapor generation quantitatively, four distinct Janus-structured evaporators were tested under 1 sun illumination (Figure 4c and Figure S9). The decline in water mass under continuous solar radiation exhibits a linear trend across all tested evaporators, with Janus-1.0 achieving the highest evaporation rate of 2.26 kg m^−2^ h^−1^, approximately five times greater than that of pure water (0.46 kg m^−2^ h^−1^). Among these Janus-structured evaporators, the lower evaporation rate of Janus-0 is likely associated with its reduced light absorption capability. While Janus-1.5 and Janus-1.0 exhibit similar light absorption properties, the thicker hydrophobic layer in Janus-1.5 hinders vapor escape [47], slightly compromising its rate. Therefore, the superior performance of Janus-1.0 can be attributed to its balanced hydrophobic and hydrophilic design.

Solar evaporation efficiency (*η*) is calculated using Equations (1) and (2):

*η* = *ṁ*Δ*H_vap_/P*_0_ (1)

Δ*H_vap_* = Δ*H_PF_,_T1_ + C_W_(T_I_ − T_S_)* (2)

where *ṁ* represents the net evaporation rate (subtracting natural evaporation, Figure S10), Δ*H_vap_* is the total enthalpy of sensible heat (*C_W_*(*T_I_ – T_S_*)) and the equivalent enthalpy of the water (Δ*H_PF_,_T_*_1_) (Note S2), *C_W_* denotes the specific heat capacity of water, *T_I_* represents the surface temperature of the evaporator, *T_S_* is the initial temperature of the water, and *P*_0_ is the solar irradiation intensity (1000 W m^−2^). The *η* value for Janus-1.0 reached 84.6% (Figure 4d), surpassing those of Janus-0 (72.0%), Janus-0.5 (78.6%), and Janus-1.5 (81.7%). In addition, the Janus-1.0 evaporator demonstrates the highest dark evaporation rate of 0.56 kg m^−2^ h^−1^, which can be attributed to its enthalpy of evaporation (Figure S11). It is well established that hydrogels can effectively alter the state of water [21,48,49]. To investigate the O-H stretching behavior of water within the evaporator, Raman spectroscopy was employed. It is well established that water molecules exhibit distinct interaction states with hydrophilic materials, commonly categorized as bound water (BW), intermediate water (IW), and free water (FW). Among these, FW behaves similar to bulk water with negligible interfacial interactions, whereas IW occupies an intermediate state characterized by partially disrupted hydrogen bonds. The IW/FW ratio serves as a critical performance indicator because the reduced hydrogen-bonding network of IW lowers the theoretical evaporation enthalpy compared to FW, thereby enhancing evaporation kinetics under photothermal conditions. As shown in Figure 4e, the peaks at approximately 3260 cm^−1^ and 3401 cm^−1^ are assigned to FW, while the peaks at 3508 cm^−1^ and 3612 cm^−1^ correspond to IW. By fitting the Raman spectra, the ratio of IW to FW in pure water is determined to be 0.23, whereas this ratio increases significantly to 0.85 in the evaporator. This enhanced IW/FW ratio effectively accelerates the water evaporation process. The stability of the Janus-structured evaporators was further examined through continuous 12 h evaporation tests (Figure 4f). All samples maintained stable evaporation rates over 12 h, with no significant performance degradation, confirming the robustness of the Janus design.

### 2.4. Evaporation Performance for Brine

**Figure 5:**
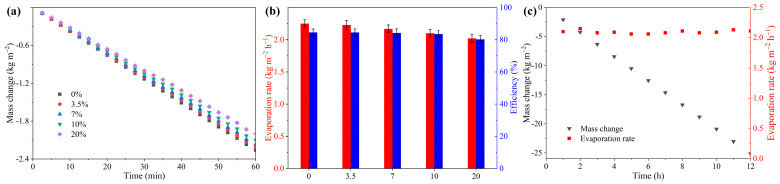
**Evaporation performance of the Janus-structured evaporator for brine desalination.** (**a**) Mass loss profiles of brines with varying NaCl concentrations under simulated AM 1.5G spectral illumination. (**b**) Comparison of evaporation rates and corresponding efficiencies for brines of different salt concentrations. (**c**) Stability test of the Janus-structured evaporator during prolonged operation with 10% brine.

The evaporation performance of brine solutions was investigated using the Janus-1.0 evaporator under varying NaCl concentrations. Figure 5a illustrates the weight loss curves for brine solutions of different concentrations, revealing that the evaporation rate for low-concentration brine is comparable to that of pure water. Notably, even at high salt concentrations, the system maintains effective performance, with an evaporation rate reaching 2.10 kg m^−2^ h^−1^ at 10% NaCl concentration. A slight decline in the evaporation rate is observed as salt concentration increases. However, the system demonstrates remarkable robustness, sustaining a high evaporation rate of 2.00 kg m^−2^ h^−1^ even at 20% salinity. To understand the impact of salinity, the energy conversion efficiencies of brine solutions with 3.5%, 7%, 10%, and 20% NaCl concentrations were calculated based on energy loss analysis (Note S3). Figure 5b demonstrates that the evaporation efficiencies for these concentrations are 84.5%, 84.2%, 83.5%, and 80.2%, respectively. These findings indicate that the salt content has a negligible impact on the photothermal evaporation performance of the Janus-structured evaporator. Importantly, the observed evaporation performance matches or exceeds the performance of state-of-the-art evaporators featuring Janus architectures (Table S1).

A critical challenge in solar desalination lies in managing salt accumulation, which can obstruct water flow, reduce light absorption, and hinder vapor escape, thereby lowering evaporation rates. The innovative Janus design effectively addresses these issues through its unique double-layer structure and unidirectional flow system. The hydrophilic PVA-CF@TA-Fe^3+^ layer facilitates efficient water transport during evaporation, while the hydrophobic PVDF-HFP@PPy layer and the unidirectional flow ensure efficient vapor escape and prevent salt accumulation within the evaporator. The long-term desalination capability of the Janus-structured evaporator is further validated through a 12 h experiment using a 10% brine solution under solar irradiation, as shown in Figure 5c and Figure S12. Due to the synergistic unidirectional flow and Janus structural design, the evaporator maintains stable and efficient evaporation (Figure 5c) without detectable salt precipitation on its surface (Figure S12). To validate long-term stability, 5-day continuous evaporation tests were conducted, confirming consistent evaporation performance (Figure S13). These results position it as a promising candidate for real-world desalination applications.

### 2.5. Outdoor Evaporation and Wastewater Purification Performance

**Figure 6:**
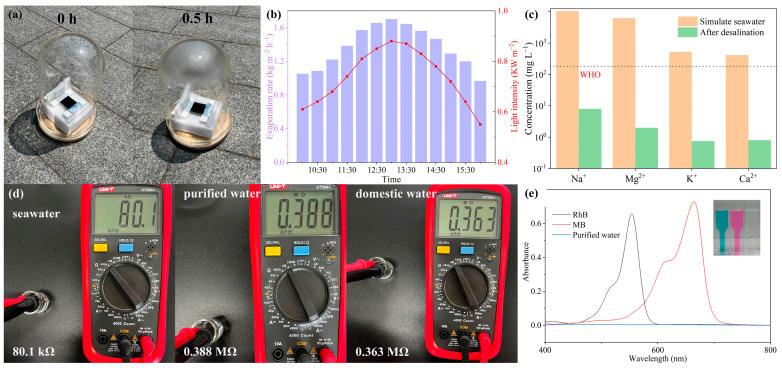
Outdoor evaporation testing and wastewater purification performance. (**a**) Photograph of the outdoor experimental setup used to simulate seawater evaporation under natural sunlight. (**b**) Evaporation rate of the Janus-structured evaporator measured at different times of the day (10:00 to 16:00) under varying light intensity levels. (**c**) Variations in major ion concentrations before and after the evaporation process. (**d**) Electrical resistivity of the purified water measured using a multimeter. (**e**) UV-Vis absorption spectra of Rhodamine-B (RhB) and Methylene Blue (MB) dye solutions before and after treatment with the evaporator.

To evaluate the performance of the Janus-structured evaporator under real-world conditions, an outdoor evaporation test was conducted on a sunny day. The experimental setup consisted of a glass enclosure, a foam platform, and a small water reservoir (Figure 6a and Figure S14). The apparatus was placed on a rooftop with unobstructed sunlight to ensure optimal illumination. A simulated seawater solution containing 26.726 g/L NaCl, 22.26 g/L MgCl_2_, 3.248 g/L MgSO_4_, 1.153 g/L CaCl_2_, and 1 g/L KCl was prepared to monitor changes in ionic concentrations before and after the evaporation process. During the evaporation process, water vapor condenses on the inner surface of the glass cover and is collected as droplets. The results of the outdoor evaporation test are shown in Figure 6b–d. The data reveal that light intensity peaked at approximately 13:00, achieving an evaporation rate of 1.70 kg m^−2^ h^−1^ at 0.88 sun intensity. The diminished evaporation performance relative to indoor simulations stems from three factors: (1) evaporation suppression under elevated humidity conditions within the enclosed system, (2) reduced incident light intensity, and (3) parasitic light attenuation caused by vapor condensation at the upper dome surfaces. The ionic concentrations in the collected condensate are significantly reduced compared to the simulated seawater. The concentrations of Na^+^, Mg^2+^, K^+^, and Ca^2+^ decreased by 3 orders of magnitude from initial levels of 10,507 mg/L, 6274 mg/L, 524 mg/L, and 416 mg/L to 8 mg/L, 2 mg/L, 0.8 mg/L, and 0.8 mg/L, respectively, meeting the WHO standards for drinking water quality [48,50]. Remarkably, under sufficient illumination, an evaporator with an area of 1 m^2^ can produce an amount of purified water equivalent to an adult’s daily water requirement in just 1 h.

In addition to desalination, the potential of the Janus-structured evaporator to treat industrial dye wastewater was explored. A simulated wastewater solution containing 10 mg/L of Rhodamine-B (RhB) and 5mg/L of Methylene Blue (MB) was tested. As shown in Figure 6e, UV-Vis spectroscopy analysis of the water before and after purification, along with photographic evidence, demonstrated complete removal of organic dyes. This confirms the capability of the evaporator to transform seawater and select industrial wastewater into high-purity potable water. These results highlight the practicality and effectiveness of the evaporator as a solar interfacial evaporation technology for addressing global challenges in desalination and wastewater treatment.

## 3. Conclusions

In summary, a novel Janus-structured evaporator combining a hydrophobic PVDF-HFP@PPy membrane with a hydrophilic PVA-CF@TA-Fe^3+^ hydrogel was successfully developed. The evaporators demonstrated superior photothermal conversion efficiency, achieving over 90% absorbance within the standard solar spectrum (AM 1.5 G). The incorporation of a hydrophilic and porous PVA-CF@TA-Fe^3^⁺ hydrogel played a pivotal role in ensuring a continuous water supply and activating water molecules at the evaporation interface, which significantly reduced the enthalpy of water evaporation. This synergistic design enabled dual vapor escape pathways under both unidirectional flow and suspension modes, achieving a maximum evaporation rate of 2.26 kg m^−2^ h^−1^ with a solar-to-vapor conversion efficiency of 84.6% under 1 sun illumination at an optimal suspension height of 1 cm. Moreover, the dual anti-salt strategies effectively mitigated salt accumulation, maintaining stable performance under high-salinity conditions. During prolonged desalination of 10% brine, the evaporator sustained an evaporation rate of 2.10 kg m^−2^ h^−1^ without observable salt crystallization, confirming its robustness and long-term durability. Outdoor performance tests and dye wastewater treatment further confirmed the potential of this technology for seawater desalination under natural sunlight and contaminated water purification. Furthermore, the evaporator’s simple and scalable fabrication process, combined with low-cost, widely available raw materials, enables cost-effective large-scale manufacturing. Critically, the hydrophobic PVDF-HFP@PPy layer exhibits inherent non-fouling characteristics, which resist adhesion to ionic contaminants. Concurrently, the physically crosslinked PVA-CF@TA-Fe^3+^ hydrogel eliminates chemical crosslinker requirements while offering closed-loop recyclability, and the spatially decoupled unidirectional flow design allows modular scale-up without compromising evaporation efficiency—paving the way for industrial deployment of sustainable seawater desalination.

## 4. Materials and Methods

### 4.1. Materials

Polyvinyl alcohol (PVA), polyvinylidene fluoride-hexafluoropropylene (PVDF-HFP), copper acetate, tetrahydrofuran (THF), N,N-dimethylformamide (DMF), pyrrole (Py), ferric nitrate (Fe(NO_3_)_3_·9H_2_O), and tannic acid (TA) were obtained from Aladdin Reagents Co, Ltd. Deionized water was prepared in the laboratory following a standard protocol to ensure purity. The cellulose fabric and rice paper were sourced from local suppliers. All chemicals used in this study were of analytical grade and were utilized without any additional purification.

### 4.2. Preparation of PVA–Cellulose Fabric (PVA-CF)

The cellulose fabric (CF) was initially immersed in anhydrous ethanol, thoroughly cleaned by repetitive dipping and washing cycles, and subsequently dried at 40 °C to obtain a clean CF. To prepare a 5% aqueous solution of PVA, 0.5 g of PVA was dissolved in 9.5 g of deionized water under continuous magnetic stirring at 90 °C in a water bath for 3 h to ensure complete dissolution. After cooling to room temperature, the PVA solution was uniformly applied to the treated CF. Excess PVA was then removed using a precision film scraper set to 250 μm to control the sample thickness.

### 4.3. Preparation of PVDF-HFP@PPy Nanofiber Membrane

To synthesize the PVDF-HFP@PPy membrane, 1.5 g of PVDF-HFP was weighed and transferred into a glass vial, followed by the addition of 0.3 g of copper acetate. Subsequently, 3 mL of DMF and 3 mL of THF were sequentially added to the vial. The mixture was stirred at 30 °C until complete dissolution to form a homogenous electrospinning solution. The PVDF-HFP nanofiber membrane was fabricated via electrospinning under the following parameters: an applied voltage of 18 kV, a feed rate of 1 mL/h, and a working distance (needle-to-collector) of 20 cm. The fibers were directly deposited onto rice paper, forming a continuous and uniform nanofibrous layer. To facilitate PPy synthesis, the PVDF-HFP membrane was exposed to a high-temperature reaction at 90 °C for 3 h, during which Py vapor was polymerized under oxidative conditions induced by Cu^2+^ ions. This process resulted in uniform PPy deposition on the PVDF-HFP nanofibers, endowing the membrane with photothermal conversion properties.

### 4.4. Preparation of the Janus-Structured Evaporator

The Janus-structured material was constructed by laminating the PVDF-HFP@PPy nanofiber membrane onto the PVA-coated cellulose fabric. The composite was subjected to a freeze–thaw crosslinking process, where it was frozen at −20 °C for 8 h and thawed at room temperature for 3 h, and the cycle was repeated 3 times. The resulting material was rinsed with deionized water before being floated in a 1 mg/mL TA solution for 6 h, followed by washing and subsequent floating in a 20 mg/mL Fe^3+^ solution for 10 min to facilitate chelation. This process yielded a Janus-structured photothermal evaporator featuring a hydrophobic PVDF-HFP@PPy layer and a hydrophilic PVA-CF@TA-Fe^3+^ layer. To investigate the relationship between the thickness of the hydrophobic PVDF-HFP@PPy nanofiber membrane and the evaporation rate, four evaporators with varying membrane thicknesses were prepared by varying the electrospinning solution volume (0, 0.5, 1.0, and 1.5 mL). The evaporator prepared with 0 mL of electrospinning solution, consisting solely of the PVA-CF@TA-Fe^3+^, was used as a control. These samples were denoted as Janus-0, Janus-0.5, Janus-1.0, and Janus-1.5, respectively, to facilitate the comparison of evaporation performance.

### 4.5. Characterization

The surface morphology of the Janus-structured evaporators was investigated using a scanning electron microscope (SEM, FlexSEM 1000, Hitachi, Tokyo, Japan). X-ray diffraction (XRD) patterns were recorded with an X-ray diffractometer (Smartlab SE, Rigaku, Tokyo, Japan) in the range of 10° to 70° at a scanning speed of 3°/min. The chemical structure of the evaporators was evaluated using a Thermo FTIR spectrometer (Nicolet is50, Thermo Fisher Scientific, Waltham, MA, USA). Raman spectra were collected with a spectrometer (InVia Qontor, Renishaw, Wotton-under-Edge, UK) at an excitation wavelength of 532 nm. The optical properties of the photothermal layer were characterized by reflectance spectra (200–2500 nm) using a UV-Vis-NIR spectrophotometer (Lambda 950, PE, Waltham, MA, USA). The wetting properties of the Janus-structured evaporator were quantified by measuring the contact angles of both surfaces using a contact angle meter (C20, Kono, Boston, MA, USA). An infrared thermal imager (E60, FLIR, Wilsonville, OR, USA) was used to monitor the surface temperature distribution of the samples under solar illumination.

### 4.6. Evaporation Measurements

To measure evaporation performance, a test sample measuring 1 cm × 4 cm was positioned horizontally between two foam pieces. A capillary wicking belt was connected to both ends of the sample, facilitating controlled water transport. One end of the belt was linked to a syringe pump calibrated to extrude water at a constant rate of 1 mL/h, while the other end was attached to a vial placed on a precision electronic scale (BSA224s-CW, resolution 0.1 mg) to record the mass of the effluent. The effective evaporation area was limited to a central 1 cm × 2 cm section of the sample, with the remaining ends shielded by aluminum foil to eliminate evaporation from inactive zones. The test was conducted under a solar simulator (MC-XS500, Merry Change, Beijing, China) with a calibrated radiation intensity of 1.0 kW/m^2^, verified using a photopower meter (VLP-2000). The experiments were conducted in a controlled laboratory environment, maintained at a temperature of 25 ± 1 °C and a relative humidity of 50 ± 5%. A Pt100 thermal resistance sensor was used to record the steam temperature during evaporation. The evaporation rate was calculated from the difference in mass between inflow and outflow water, normalized to the effective evaporation area. Each sample was allowed to stabilize under 1 sun illumination for 30 min before evaporation rates were measured, ensuring reliable and reproducible results.

## Data Availability

Data will be made available on request.

## Supplementary Materials

The following supporting information can be downloaded at: https://www.mdpi.com/article/10.3390/gels11050368/s1, Figure S1: SEM images of the PVDF-HFP membrane: (a) low magnification and (b) high magnification; Figure S2: SEM images of cross-section of Janus-structured evaporator: (a) Jauns-0, (b) Janus-0.5, (c) Janus-1.0 and (d) Janus-1.5; Figure S3: SEM images of the cellulose fabric: (a) low magnification and (b) high magnification; Figure S4: Evolution of water contact angles over time for PVA-CF membrane; Figure S5: Evolution of water contact angles over time for PVDF-HFP membrane; Figure S6: Photographs of PVDF-HFP@PPy hydrophobic membranes and cellulose fabric: (a) hydrophobic membranes of varying thicknesses; (b) cellulose fabric; Figure S7: Evolution of surface temperature for the Janus-1.0 evaporator and bulk water during exposure to one sun illumination; Figure S8: Infrared thermal images of the suspended Janus-1.0 evaporator under one sun irradiation; Figure S9: Photograph of unidirectional fluid evaporation device; Figure S10: The mass change curves as a function of time for water in the Janus-structured evaporator and bulk water in the dark; Figure S11: The enthalpy of evaporation of the Janus-structured evaporator and bulk water; Figure S12: Infrared thermal images of salt water evaporation in a Janus-structured evaporator; Figure S13: Average evaporation rate of 10% brine over time; Figure S14: Photographs of outdoor evaporation device; Table S1: Evaporation rates of Janus-structured evaporators with different designs; Note S1: Calculation of the effective absorption; Note S2: Evaluation of evaporation enthalpy of water in Janus evaporator; Note S3: Analysis of heat loss. References [34,51,52,53,54,55,56,57,58,59,60,61,62,63,64,65] are cited in the Supplementary Materials.
