# Supplementary material for: Preparation of Janus-Structured Evaporators for Enhanced Solar-Driven Interfacial Evaporation and Seawater Desalination
Source: Gels. 2025 May 17;11(5):368. doi: 10.3390/gels11050368 (PMC12111585; doi:10.3390/gels11050368)
Supplement: Supplementary file 1 [file gels-11-00368-s001.zip › gels-3620269-supplementary.pdf]

Supplementary Materials

Preparation of Janus-Structured Evaporators for Enhanced Solar-Driven Interfacial Evaporation and Seawater Desalination

Junjie Liao [1,2], Luyang Hu [1,2,*], Haoran Wang [1], Zhe Yang [2], Xiaonan Wu [2] and Yumin Zhang [3]

1 School of Materials Science and Engineering, Anhui University of Science and Technology,
Huainan 232001, China; 17355451848@163.com (J.L.); hrwang2013@163.com (H.W.)
2 Anhui Industrial Generic Technology Research Center for New Materials from Coal-based Solid Wastes, Huainan 232001, China; yangzhe010120@163.com (Z.Y.); 13839318581@163.com (X.W.)
3 National Key Laboratory of Science and Technology on Advanced Composites in Special Environment, Harbin 150001, China; yuminzhang@163.com
* Correspondence: huluyang@gmail.com

Note S1. Calculation of the effective absorption

Reflectance spectra spanning 200-2500 nm for the Janus-structured evaporators were obtained using a UV-Vis-NIR spectrophotometer (PE Lambda 950) equipped with an integrating sphere unit. To comprehensively evaluate the light absorption properties of the evaporators, the light absorption rate (α) was calculated using equation S1:

$$\alpha = \frac{\int_{200}^{2500} I_{AM1.5}(\lambda)(1 - R(\lambda))\mathrm{d}\lambda}{\int_{200}^{2500} I_{AM1.5}(\lambda)\mathrm{d}\lambda} \times 100\% \tag{S1}$$

In this equation, $R(\lambda)$ represents the reflectivity of the sample at a given wavelength λ, and $I_{AM1.5}(\lambda)$ corresponds to the illumination intensity of the AM 1.5 solar spectrum at the same wavelength. The absorbance values for different variations of the evaporator, namely Janus-0, Janus-0.5, Janus-1.0, and Janus-1.5, were determined to be 80.4%, 90.1%, 91.4%, and 89.5%, respectively.

Note S2. Evaluation of evaporation enthalpy of water in Janus evaporator

The enthalpy of evaporation was determined based on equivalent calculations described in prior studies [1-3]. Experiments were conducted in a closed dryer, where bulk water and the Janus-structured evaporators were placed. The evaporators were suspended using a foam device to ensure identical surface areas on both the top and bottom sides, enabling dark evaporation under controlled conditions. The enthalpy of evaporation for water in the evaporator (E_{PF}) is defined using Equation (S2):

$$U_{in} = 2E_{BW}m_{BW} = E_{PF}m_{PF} \tag{S2}$$

Here, E_{BW} represents the evaporation enthalpy of bulk water, calculated using the empirical formula $1.91846 \times 10^3 \times T_0^2/(T_0 - 33.91)^2$ J/g, where T_0 denotes the surface temperature of the sample. m_{BW} and m_{PF} are the mass changes of bulk water and water in the Janus-structured evaporator under dark conditions at room temperature, respectively [4].

To correct the evaporation enthalpy (ΔH_{PF}) of water within the evaporator for its surface temperature (T_I) under illumination, Equation (S3) was applied:

$$\Delta H_{PF,T1} = C_w(298.15 - T_I) + \Delta H_{PF,298.15K} + C_v(T_I - 298.15) \tag{S3}$$

Here, C_w and C_v denote the specific heat capacities of water in the liquid and gaseous states, respectively. $\Delta H_{PF,298.15K}$ represents the evaporation enthalpy at 298.15 K. The total enthalpy of vaporization (ΔH_{vap}), which encompasses both the sensible heat and the equivalent enthalpy of water, was calculated using Equation (S4):

$$\Delta H_{vap} = \Delta H_{PF,T1} + C_W(T_I - T_S) \tag{S4}$$

In this equation, C_W is the specific heat capacity of bulk water, and T_S is the initial temperature of the water.

Note S3. Analysis of heat loss

The thermal energy losses of the Janus-structured evaporator were systematically quantified through radiation loss, convection loss, and transmission heat loss. The Stefan–Boltzmann equation (S5) was employed to calculate the radiation loss (Φ), where the emissivity (ε) of the evaporator was assumed to be 0.96. The surface area (A) was approximately ~ 2 cm², and the Stefan–Boltzmann constant (σ) was 5.67×10^{-8} W m^{-2} K^{-4}.

For a Janus-structured evaporator with a surface temperature (T_I) of 33.5 °C and upper (T_u) and lower (T_l) air temperatures both at 30.5 °C, the radiation loss is calculated as 7.42×10^{-3} W. When the suspension height is 0 cm, only the upper surface is considered for thermal radiation loss.

$$\Phi = \varepsilon A\sigma[(T_I^4 - T_u^4) + (T_I^4 - T_l^4)] \quad \text{(S5)}$$

Heat convection loss (Q_1) was determined using Newton's cooling law (S6), where the natural convective heat transfer coefficient (h) was set to 5 W m^{-2} K^{-1}. Under these conditions, Q_1 was estimated to be 6.0×10^{-3} W.

$$Q_1 = hA[(T_I - T_u) + (T_I - T_l)] \quad \text{(S6)}$$

Transmission heat loss (Q_2) was calculated using formula (S7), where C_F represents the specific heat capacity of the fluid, m is the fluid mass flow rate, and ΔT is the temperature difference between the fluid outlet and its initial state. For pure water evaporation at a suspension height of 1 cm, ΔT was measured at 8.5 °C, resulting in Q_2 being 5.43×10^{-3} W.

$$Q_2 = C_F m\Delta T \quad \text{(S7)}$$

The total energy loss rate (χ) was then computed using equation (S8), accounting for the

combined losses from radiation (Φ), convection (Q_1), and transmission (Q_2). For the Janus-1.0 membrane, with an absorption efficiency (α) and incident solar power (P_0), χ was calculated as 18%.

$$\chi = 1 - \alpha + (\Phi + Q_1 + Q_2)/(AP_0) \tag{S8}$$

Comparative analysis of the energy loss rates for different membrane configurations—Janus-0, Janus-0.5, Janus-1.0, and Janus-1.5—revealed values of 28.8%, 19.1%, 18.0%, and 19.7%, respectively. The lowest loss rate observed for Janus-1.0 underscores the importance of optimizing suspension height and membrane design for minimizing energy dissipation and enhancing overall efficiency.

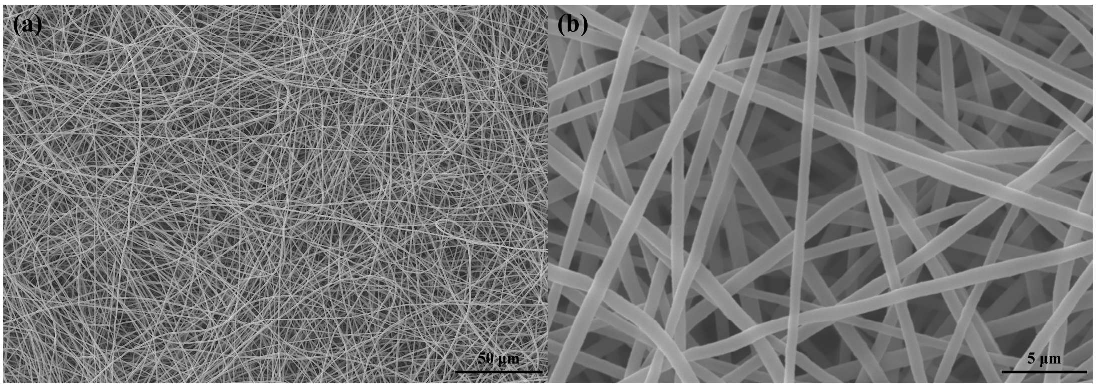

Figure S1. SEM images of the PVDF-HFP membrane: (a) low magnification and (b) high magnification.

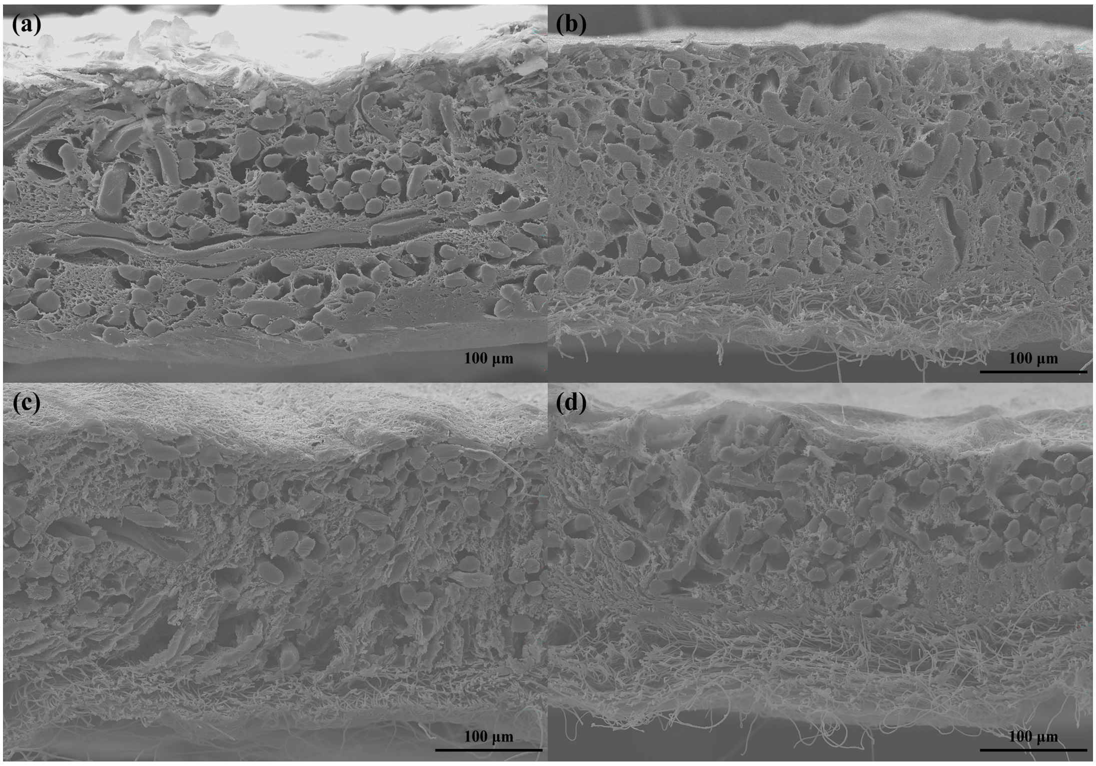

Figure S2. SEM images of cross-section of Janus-structured evaporator: (a) Janus-0, (b) Janus-0.5, (c) Janus-1.0 and (d) Janus-1.5.

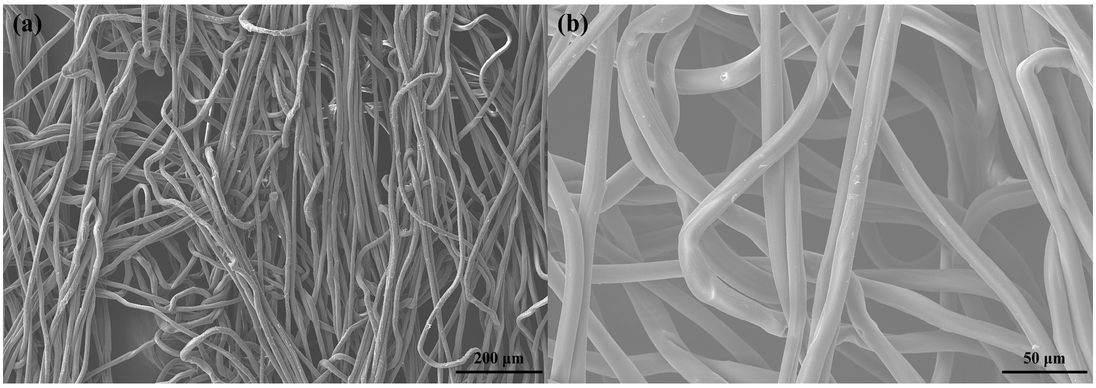

Figure S3. SEM images of the cellulose fabric: (a) low magnification and (b) high magnification.

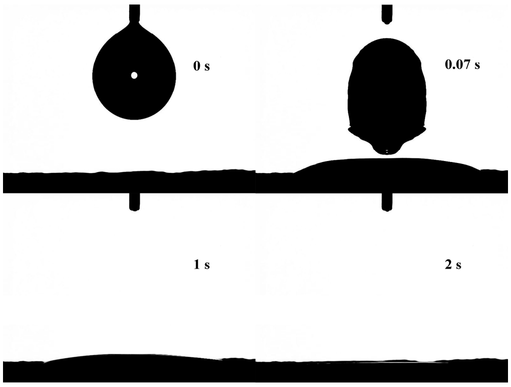

Figure S4. Evolution of water contact angles over time for PVA-CF membrane.

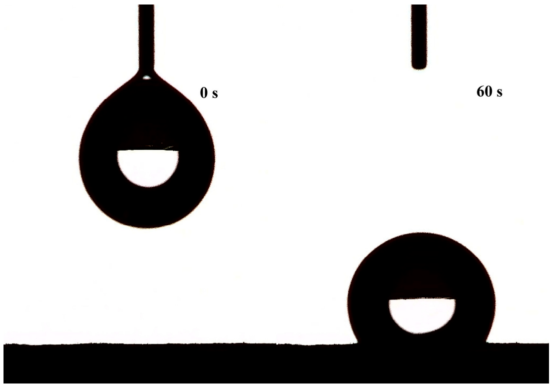

Figure S5. Evolution of water contact angles over time for PVDF-HFP membrane.

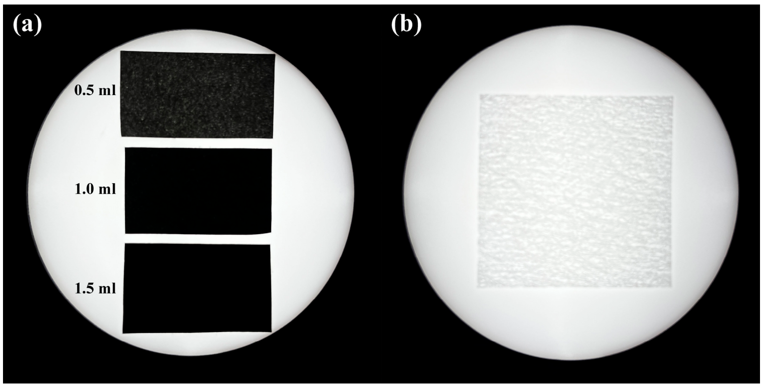

Figure S6. Photographs of PVDF-HFP@PPy hydrophobic membranes and cellulose fabric: (a) hydrophobic membranes of varying thicknesses; (b) cellulose fabric.

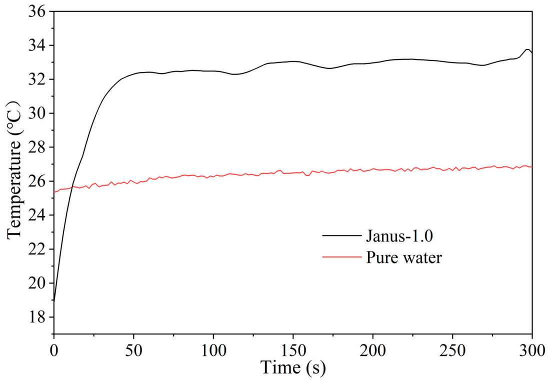

Figure S7. Evolution of surface temperature for the Janus-1.0 evaporator and bulk water during exposure to one sun illumination.

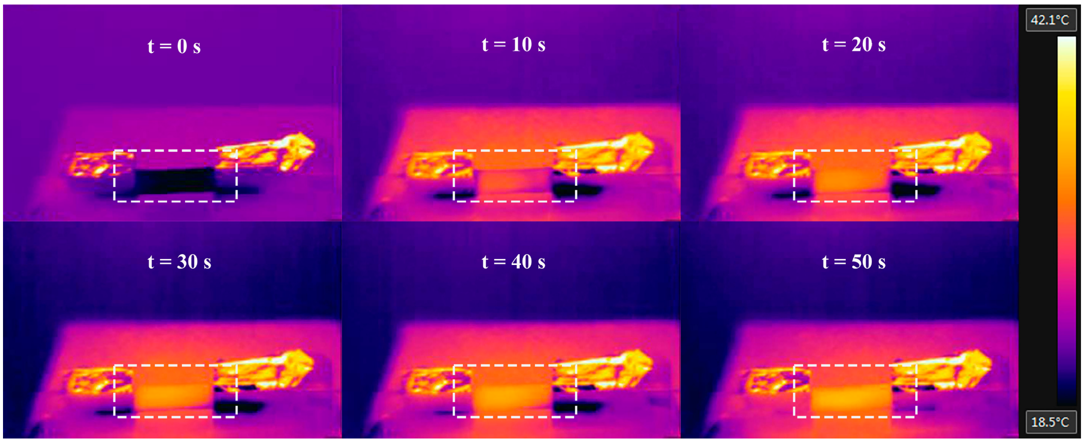

Figure S8. Infrared thermal images of the suspended Janus-1.0 evaporator under one sun irradiation.

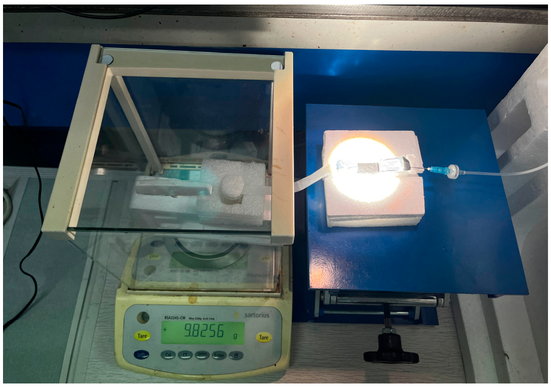

Figure S9. Photograph of unidirectional fluid evaporation device.

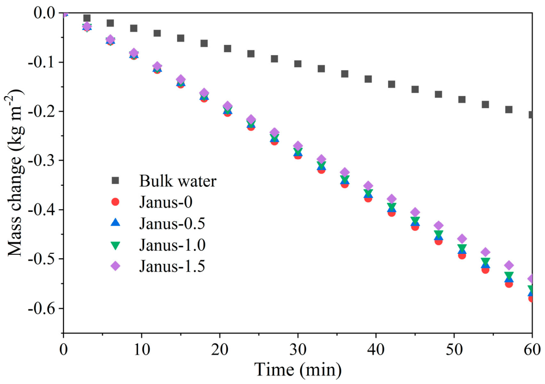

Figure S10. The mass change curves as a function of time for water in the Janus-structured evaporator and bulk water in the dark.

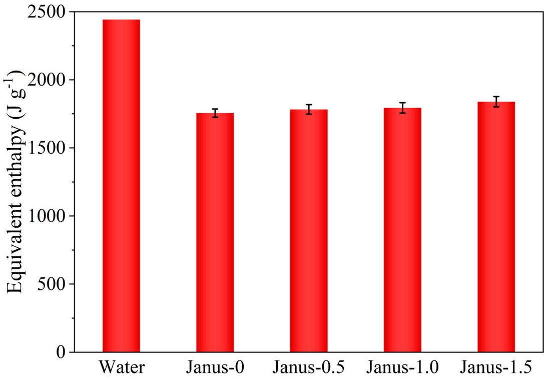

Figure S11. The enthalpy of evaporation of the Janus-structured evaporator and bulk water.

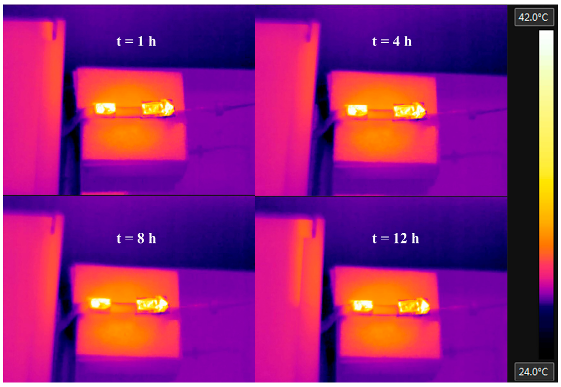

Figure S12. Infrared thermal images of salt water evaporation in a Janus-structured evaporator.

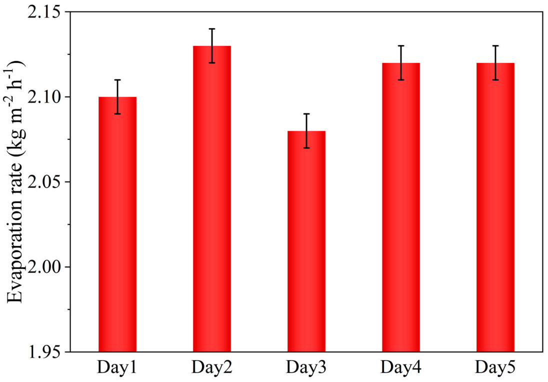

Figure S13. Average evaporation rate of 10% brine over time.

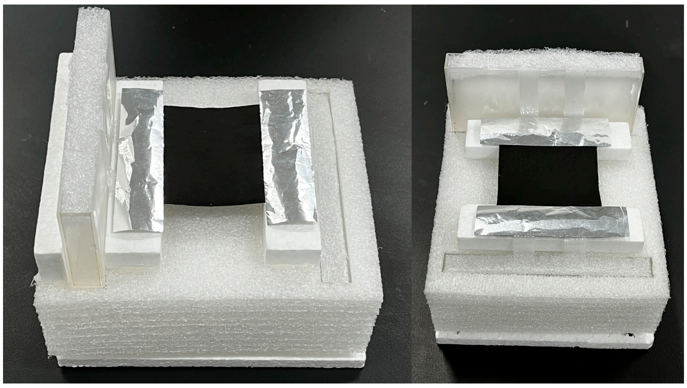

Figure S14. Photographs of outdoor evaporation device.

Table S1. Evaporation rates of Janus-structured evaporators with different designs.

Material	Brine concentration (wt.%)	$\dot{m}$ (kg m^{-2} h^{-1})	Reference
PZCA membrane	0	1.31	[5]
Asymmetric polypyrrole membrane	0	2.03	[6]
	3.5-10	2.00-1.85	
PDMS/Si-NaOH	0	2.18	[7]
	3.5	2.17	
	10	2.10	
PPG@PU/ PDMS@PU-CNTs	3.5	1.34	[8]
Co@C/NCNT photothermal membrane	0	1.55	[9]
TiN/GO film	0	1.7	[10]
Nanofibers/rGO	0	1.83	[11]
JPCF	0-3.5	1.77-1.75	[12]
C@CuO membrane	0	1.88	[13]
SiO_2/MXene/Poly tetrafluoroethylene membrane	0	1.53	[14]
SiO_2/ CNT@epoxy	0	1.84	[15]
PPy@Ni sponge	0	1.71	[16]
Janus-structured evaporator	0	2.26	This work
	3.5-20	2.22-2.00	

References

[1] Zhao, F.; Zhou, X.; Shi, Y.; Qian, X.; Alexander, M.; Zhao, X.; Mendez, S.; Yang, R.; Qu, L.; Yu, G.; Highly efficient solar vapour generation via hierarchically nanostructured gels. *Nat. Nanotechnol.* **2018**, 13 489-495.

[2] Liu, H.; Chen, B.; Chen, Y.; Zhou, M.; Tian, F.; Li, Y.; Jiang, J.; Zhai, W. Bioinspired self-standing, self-floating 3D solar evaporators breaking the trade-off between salt cycle and heat localization for continuous seawater desalination. *Adv. Mater.* **2023**, 35, 2301596.

[3] Zhang, F.; Li, Y.; Bai, X.; Wang, S.; Liang, B.; Fu, G.; Wu, Z. Synthesis of mesoporous Fe_3Si aerogel as a photo-thermal material for highly efficient and stable corrosive-water evaporation. *J. Mater. Chem. A.* **2018**, 6, 23263-23269.

[4] Yang, B.; Zhang, Z.; Liu, P.; Fu, X.; Wang, J.; Cao, Y.; Tang, R.; Du, X.; Chen, W.; Li, S.; Yan, H.; Li, Z.; Zhao, X.; Qin, G.; Chen, X.; Zuo, L. Flatband λ-Ti_3O_5 towards extraordinary solar steam generation. *Nature* **2023**, 622, 499-506.

[5] Chen, H.; Pan, G.; Yan, M.; Wang, F.; Wu, Y.; Guo, C. Janus membrane with enhanced interfacial activation for solar evaporation. *J. Energy Chem.* **2023**, 87, 1-11.

[6] Gao, C.; Li, Y.; Lan, L.; Wang, Q.; Zhou, B.; Chen, Y.; Li, J.; Guo, J.; Mao, J. Bioinspired asymmetric polypyrrole membranes with enhanced photothermal conversion for highly efficient solar evaporation. *Adv. Sci.* **2024**, 11, 2306833.

[7] Zhou, C.; Mei, Q.; Huang, L.; Mao, T.; Li, S.; Wang, Z.; Wan, H.; Gu, H.; Han, K. Flexible Janus black silicon photothermal conversion membranes for highly efficient solar-driven interfacial water purification. *ACS Appl. Mater. Interfaces.* **2024**, 16, 26153-26166.

[8] Liu, H.; Gu, J.; Liu, Y.; Yang, L.; Wang, L.; Yu, J.; Qin, X. Reconfiguration and self-healing integrated

Janus electrospinning nanofiber membranes for durable seawater desalination. *Nano Res.* **2022**, 16, 489-495.

[9] Jiang, J.; Jiang, H.; Xu, Y.; Chen, M.; Ai, L. Janus Co@C/NCNT photothermal membrane with multiple optical absorption for highly efficient solar water evaporation and wastewater purification. *Colloids Surf. A Physicochem. Eng. Aspects.* **2022**, 647, 128960.

[10] Du, Y.; Wen, J.; Deng, K.; Zou, L.; Liu, X.; Liu, P.; Liu, B.; Lv, X.; Tian, W.; Ji, J. Janus film evaporator with improved light-trapping and gradient interfacial hydrophilicity toward sustainable solar-driven desalination and purification. *Sep. Purif. Technol.* **2023**, 322, 124312.

[11] Sui, Z.; Xue, X.; Wang, Q.; Li, M.; Zou, Y.; Zhang, W.; Lu, C. Facile fabrication of 3D Janus foams of electrospun cellulose nanofibers/rGO for high efficiency solar interface evaporation. *Carbohydr. Polym.* **2024**, 331, 121859.

[12] Sun, J.; Xin, Y.; Li, Z.; Sun, B.; Fan, X.; Rapid preparation of Janus biomass evaporator by dielectric barrier discharge plasma for high-efficiency desalination and wastewater purification. *Chem. Eng. J.* **2024**, 484, 149669.

[13] Hou, L.; Wang, N.; Yu, L.; Liu, J.; Zhang, S.; Cui, Z.; Li, S.; Li, H.; Liu, X.; Jiang, L. High-Performance Janus solar evaporator for water purification with broad spectrum absorption and ultralow heat loss. *ACS Energy Lett.* **2023**, 8, 553-564.

[14] Li, H.; Li, L.; Xiong, L.; Wang, B.; Wang, G.; Ma, S.; Han, X. SiO_2/MXene/poly(tetrafluoroethylene)-based Janus membranes as solar absorbers for solar steam generation. *ACS Appl. Nano Mater.* **2021**, 4, 14274-14284.

[15] Tan, X.; Cheng, Y.; Wang, S. Design of interface-stable Janus solar-energy evaporator. *Int. J. Therm. Sci.* **2022**, 179, 107712.

[16] Song, R.; Zhang, N.; Wang, P.; Ding, H.; Wang, J.; Li, S. A self-floating Janus PPy@Ni sponge salt-resisting solar evaporator for efficient interfacial evaporation. *Appl. Surf. Sci.* **2023**, 616, 156448.
